# Omega-3 fatty acids in heart disease—why accurately measured levels matter

**DOI:** 10.1007/s12471-023-01759-2

**Published:** 2023-02-16

**Authors:** C. von Schacky, R. S. Kuipers, H. Pijl, F. A. J. Muskiet, D. E. Grobbee

**Affiliations:** 1 Omegametrix, Martinsried, Germany; 2 Heart Centre, Onze Lieve Vrouwe Gasthuis, Amsterdam, The Netherlands; 3 Department of Cardiology, Dijklander Hospital, Purmerend/Hoorn, The Netherlands; 4 Department of Internal Medicine, Leiden University Medical Centre, Leiden, The Netherlands; 5 Department of Laboratory Medicine, University Medical Centre Groningen, University of Groningen, Groningen, The Netherlands; 6 Julius Global Health, Julius Centre for Health Sciences and Primary Care, University Medical Centre Utrecht, Utrecht, The Netherlands

**Keywords:** Cardiology, Cardiovascular Disease, Eicosapentaenoic Acid, Docosahexaenoic Acid, Omega‑3 Index, Fatty Acids

## Abstract

Current guidelines barely support marine omega‑3 fatty acids eicosapentaenoic acid (EPA) and docosahexaenoic acid (DHA) in cardiology, mainly because results of large trials were equivocal. Most large trials have tested EPA alone or EPA + DHA combined as a drug, thereby disregarding the relevance of their blood levels. These levels are frequently assessed with the Omega‑3 Index (percentage of EPA + DHA in erythrocytes), which is determined using a specific standardised analytical procedure. EPA and DHA are present in every human being at unpredictable levels (even in the absence of intake), and their bioavailability is complex. Both facts need to be incorporated into trial design and should direct clinical use of EPA and DHA. An Omega‑3 Index in the target range of 8–11% is associated with lower total mortality, fewer major adverse cardiac and other cardiovascular events. Moreover, functions of organs such as the brain benefit from an Omega‑3 Index in the target range, while untoward effects, such as bleeding or atrial fibrillation, are minimised. In pertinent intervention trials, several organ functions were improved, with improvements correlating with the Omega‑3 Index. Thus, the Omega‑3 Index is relevant in trial design and clinical medicine, which calls for a widely available standardised analytical procedure and a discussion on possible reimbursement of this test.

## Introduction

Guidelines and meta-analyses incorporate trial results using criteria for drug trials [1, 2]. Most randomised controlled double-blind intervention trials are designed as drug trials, by comparing intake of verum with placebo. However, when investigating substances present in the body at trial start, such as omega‑3 fatty acid (FA), disregarding baseline and on-trial levels becomes a methodological issue [3, 4]. This is further complicated by the complex bioavailability of lipids. Disregarding issues of bioavailability and substance levels makes such trials and their meta-analyses prone to produce erroneous results, resulting in imperfect guidelines and suboptimal clinical use of the compound.

Using the example of omega‑3 and other FAs, we herein discuss these issues and explain why their levels need to be measured in a standardised manner to better understand their actions and effects and bring their clinical use to fruition.

## Omega-3 fatty acids

### Intake versus blood levels

Two marine-derived omega‑3 FAs, eicosapentaenoic acid (EPA) and docosahexaenoic acid (DHA), are present in fatty fish and in fish, krill and algae oils. Since human metabolism of plant-derived alpha-linolenic acid (ALA) to EPA is poor and metabolism of EPA to DHA is virtually non-existent, ALA is no substitute for EPA and/or DHA [4]. Moreover, current intakes and circulating levels of EPA and DHA are probably below those on which our species has evolved and, hence, are considered optimal for human health [5, 6]. Many well-respected organisations recommend additional intake of EPA + DHA, with daily doses ranging from 200 to 4000 mg for prevention or improvement of health issues, such as cardiovascular disease or hypertriglyceridaemia, while others dispute these recommendations [1, 3, 7].

However, the bioavailability of EPA + DHA is complex. The inter-individual variability is considerable (a factor 13); concurrent intake of a fatty meal improves bioavailability by a factor of 13 (in comparison to a low-fat meal), and other influences exist [4, 8]. Thus, it is impossible to predict the bioavailability in an individual, which challenges using the same dose in everybody.

The percentage of EPA + DHA (of total FAs) measured in erythrocytes is the Omega‑3 Index. The biological variability of EPA + DHA levels in erythrocytes is low [4], and EPA + DHA levels in erythrocytes reflect those in every cell thus far studied [4]. Therefore, the Omega‑3 Index represents an individual’s EPA + DHA status.

The Omega‑3 Index is frequently determined as the percentage of EPA + DHA of a total of 26 FAs. The HS-Omega‑3 Index® is a specific analytical procedure that is supported by around 380 publications in international journals [4]. The analytical variability of this standardised and robust method is 4% [4]. It is widely used in Europe—it is identical to the method used in specific laboratories in the USA and Asia—and regularly subjected to rigorous proficiency testing [4]. Analysing the Omega‑3 Index with other methods has yielded different results [4]. Thus, while other methods may produce valid results within a study (internal validity), the standardised method produces results that are comparable with those generated using the same method in a different study (external validity)—a prerequisite for a laboratory parameter in clinical medicine.

Levels of EPA and DHA can also be measured in other sample types or FA compartments. Short-term intake is reflected in the FA of plasma, plasma phospholipids or serum, which are all used in, for example, single-dose bioavailability studies [4, 8]. With regard to clinical endpoints, such as total or cause-specific mortality or onset of type 2 diabetes mellitus, EPA + DHA levels in plasma or serum are less informative, whereas EPA + DHA levels in plasma cholesterol esters, triglycerides or adipose tissue are not informative [9, 10]. The biological variability of EPA + DHA in plasma phospholipids is larger than that in erythrocytes, making the signal-to-noise ratio for EPA + DHA in erythrocytes better than for EPA + DHA in plasma phospholipids [4]. Moreover, no standardised analytical method has been established for FAs in plasma phospholipids [4]. As discussed below, not only EPA and DHA levels but also levels of other erythrocyte FAs correlate with mortality and other health issues, suggesting the need for further research and, again, implementation of a standardised analysis. Taken together, the standardised method for determining the Omega‑3 Index and other erythrocyte FAs boasts a large scientific dataset and other important advantages, and it is therefore recommended for use in research and clinical medicine.

**Fig. 1:**
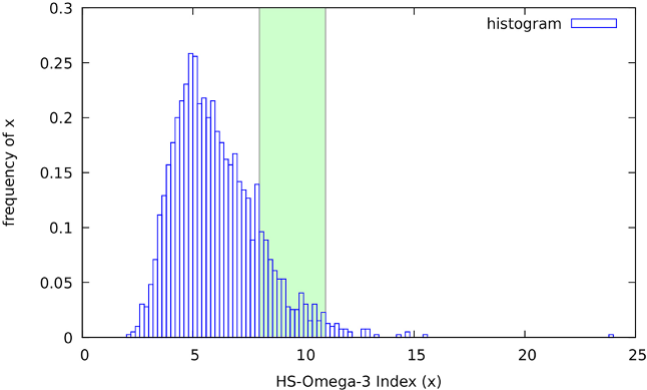
Histogram of HS-Omega‑3 Index® based on data from 1974 inhabitants of the Netherlands (only first-time measurements shown). Mean ± standard deviation is 6.06% ± 1.95%. A minority of values are within 8–11% target range (*shaded green*); few are > 11%. Concordant with previous histograms generated with this standardised method, no individuals with Omega‑3 Index < 2% were identified, indicating that human life without an Omega‑3 Index is impossible [4]. Values were derived from individuals interested in their Omega‑3 Index, who paid for analyses (non-representative sample of the Dutch population). Therefore, bias towards higher values is likely

In every population thus far studied, values of the Omega‑3 Index had a statistically normal distribution, but the means varied widely (e.g. Figure 1; [4]). For example, the mean Omega‑3 Index in patients with heart failure with reduced ejection fraction (HFrEF) or major depression is low, while it is in the target range of 8–11% in non-demented octogenarians or healthy Japanese individuals [11–13]. With the standardised method, no human being with EPA + DHA < 2% has yet been identified (Fig. 1). Specifically, such low levels have not been found in vegans who do not ingest sources of EPA + DHA, nor in athletes with a high catabolic rate [4, 14]. Thus, human life does not seem possible without a minimum value of the Omega‑3 Index.

Taken together, the facts just discussed make it non-sensical to recruit participants for a trial with EPA and/or DHA treatment without determining a baseline Omega‑3 Index. The Omega‑3 Index should be followed throughout the trial, to adjust the dose of EPA and/or DHA individually and evaluate the trial from the perspective of this index. Measurement of EPA + DHA levels in clinical routine should follow the same pattern: first, determine the need for supplementation; start with supplementation if needed; reassess the Omega‑3 Index after 3–4 months to individually adjust the dose; and perform yearly follow-ups thereafter, such as lifelong measurement of LDL cholesterol levels.

### Trial design and results

Recruiting a trial population with a high baseline Omega‑3 Index, i.e. with little or no room for improvement, will produce neutral results, as exemplified by the ASCEND trial [15]. On the other hand, recruiting a trial population irrespective of baseline levels in addition to using a low dose of EPA + DHA (e.g. somewhat less than 1 g) puts the trial at high risk of large overlaps of the omega‑3 status between the verum and placebo groups, as demonstrated by many trials with neutral results such as the SU.FOL.OM3, OMEGA, ORIGIN and VITAL studies [4].

In contrast, trials in populations with low baseline Omega‑3 Indices, such as patients with HFrEF, or trials using a high dose, such as the REDUCE-IT and JELIS trials, are more likely to show positive results [4, 11, 12, 16, 17]. In REDUCE-IT, not only was the frequency of events compared between the verum and placebo groups, but the events were also related to EPA levels reached in, in this case, serum [17, 18]. Most endpoints occurred minimally with mid-high levels, which might be comparable with an Omega‑3 Index in the target range. The primary endpoint (a combination of cardiovascular death, nonfatal myocardial infarction, nonfatal stroke, coronary revascularisation and unstable angina with hospitalisation) was reduced by 25% (when comparing verum with placebo) or by ~65% (when comparing optimal with low levels) [17, 18]. In keeping, total mortality was reduced by 13% (when comparing verum with placebo) or by 40% (when comparing optimal levels with low levels), with similar differences for stroke (28% or 50%) and all other endpoints, thus replicating epidemiological data generated with the Omega‑3 Index [17, 18].

The STRENGTH study did not show positive results, which might be partly explained by a different comparator: mineral oil in REDUCE-IT and corn oil in STRENGTH [19]. In STRENGTH, the baseline Omega‑3 Index was 5.6%, while it was not measured in REDUCE-IT [17, 20]. In the respective placebo groups, event rates in REDUCE-IT were higher than in STRENGTH [17, 19]. This leads one to speculate that a lower baseline Omega‑3 Index in REDUCE-IT than in STRENGTH might have contributed to the difference in results [17–24]. In STRENGTH, no relation of clinical events with plasma FA levels was found, illustrating the discussion above [20].

For future trials, we suggest recruiting participants who have ‘enough room for improvement’ (e.g. Omega‑3 Index < 5%), individualising EPA and DHA doses to reach the target range of 8–11% and evaluating them from the perspective of the Omega‑3 Index.

### Cardiovascular disease

In two large epidemiological studies, 10-year total mortality in cases with an Omega‑3 Index > 8% was only two thirds of the total mortality in cases with an Omega‑3 Index < 4%, with similar data reported from Framingham [21–23]. Data from Framingham show the Omega‑3 Index to be equivalent to smoking in predicting total mortality [24]. Severe clinical events, such as stroke or cardiovascular mortality, are less than half as frequent with a high Omega‑3 Index compared with a low Omega‑3 Index [23]. All findings are supported by corresponding meta-analyses, also demonstrating a lower risk of developing type 2 diabetes with higher phospholipid levels of EPA + DHA [10, 11].

Cardiovascular risk factors or other surrogate parameters, such as blood pressure, heart rate and heart rate variability, were found to improve in correlation with an increasing Omega‑3 Index [4]. LDL cholesterol subgroups change: small dense, atherogenic LDL is decreased, while large-buoyant, anti-atherogenic LDL is increased, resulting in increased LDL levels but decreased pro-atherogenic function [4]. Intermediate parameters, such as coronary lesions, unstable plaques or left ventricle remodelling after a myocardial infarction, are also improved, with improvements correlating with the Omega‑3 Index when measured [4, 25–27].

Considering the methodological issues discussed, which impede detection of the effects of EPA and DHA, it is remarkable that the latest Cochrane meta-analysis found cardiovascular mortality to be reduced by 8%, cardiovascular events by 4%, coronary heart disease mortality by 10% and coronary heart disease events by 9% (all significant), while other endpoints, such as all-cause mortality, were not found to be reduced [2]. Other meta-analyses showed similar results (e.g. [28]).

Current guidelines for cardiovascular prevention from the European Society of Cardiology (ESC) state that eating ‘fish is recommended 1–2 times a week, in particular fatty fish’, but a clear statement on EPA and DHA cannot be found [1]. Recent science advisories of the American Heart Association (AHA) were more positive towards fish and EPA + DHA in prevention of cardiovascular events [29, 30]. An omega‑3 status-based approach towards cardiovascular prevention remains to be adopted.

#### Heart failure

Low levels of EPA + DHA precede the development of HFrEF, and patients with this form of HF have a low Omega‑3 Index [11, 12, 31]. In the large trial GISSI-Heart Failure, the mean ± standard deviation (SD) Omega‑3 Index of such patients increased from 4.75% ± 1.68% to 6.73% ± 1.93% (*p* < 0.0001) after 3 months of treatment with 850–882 mg EPA + DHA per day in the verum group, below the target range of 8–11%, while it remained stable in the placebo group (4.73% ± 1.70% at baseline vs 4.81% ± 1.49% at 3 months; non-significant *p*-value) [11]. In the GISSI-Heart Failure trial, the primary endpoint (total mortality and hospitalisations combined) was significantly reduced in the verum group [32]. A recent meta-analysis found EPA + DHA supplementation to reduce rehospitalisations [33].

While previous ESC Guidelines on HFrEF included a IIb recommendation for EPA + DHA—‘An *n*-3 PUFA preparation may be considered in symptomatic HF patients to reduce the risk of cardiovascular hospitalisation and cardiovascular death’—current guidelines do not [34, 35]. The AHA Scientific Advisory Board gives a IIa recommendation and considers giving EPA + DHA to these patients ‘reasonable’ [31, 32]. Again, an omega‑3 status-based approach towards HF treatment remains to be adopted.

Contrary to HFrEF patients, the Omega‑3 Index is not low in patients with HF with preserved ejection fraction [36]. In addition, a higher Omega‑3 Index correlates with a more favourable cardiometabolic risk profile, higher aerobic capacity and lower body mass index/truncal adiposity but not with parameters of diastolic function [36].

#### Sudden cardiac death

Sudden cardiac death (SCD) is responsible for 15% of total mortality in Western countries. Automatic external defibrillators or implanted cardioverter-defibrillators only slightly reduce overall SCD events. The risk of SCD in individuals with a low Omega‑3 Index is 10 times higher than in those with a high Omega‑3 Index [4]. The incidence of SCD is increased in athletes, who are prone to low Omega‑3 Indices [4, 15]. The large GISSI-Prevenzione trial demonstrated a reduction in SCD by ~50% with 0.9 g daily EPA + DHA in patients shortly after a myocardial infarction [37].

The ESC Guidelines on the prevention of SCD ignore the results of GISSI-Prevenzione and do not mention EPA + DHA, while the AHA saw a reduction of ischaemia-induced SCD as possibly contributing to reduced mortality from coronary heart disease, resulting in their IIa recommendation for EPA + DHA [29, 30, 38, 39]. An omega‑3 status-based approach remains to be implemented.

### Other effects

The effects of EPA + DHA levels on the brain have recently been reviewed in detail [13]. Higher levels of EPA + DHA minimise brain damage by stroke (discussed above), trauma, fine particulate matter or inflammatory processes. Complex brain functions (executive function, aspects of memory or abstract thinking), correlate with the Omega‑3 Index, as do their improvements with EPA + DHA [13]. Onset of dementia is delayed and ‘age-related’ brain loss can be slowed by raising a low Omega‑3 Index [13]. Psychiatric diseases such as major or bipolar depression occur with a low Omega‑3 Index and can be improved by EPA + DHA; similar results are seen in other areas of psychopathology [13]. Other benefits have been demonstrated in and after pregnancy, in childhood and adolescence, non-alcoholic liver disease and many other instances, with improvements correlating with omega‑3 status [13, 40, 41]. Guidelines recommend EPA + DHA supplementation in pregnancy and for major depression [13, 40].

### Safety and tolerability

The European Food Safety Authority considers EPA + DHA supplementation up to 5 g daily as safe, whereas the American Food and Drug Administration considers up to 3 g daily as safe [4]. However, recently, a meta-analysis of cardiovascular intervention trials observed an increase in new-onset atrial fibrillation but not stroke[42]. The relationship between the risk of atrial fibrillation and the Omega‑3 Index is U‑shaped, with minimum risk in the Omega‑3 Index target range [43]. Moreover, bleeding events were slightly increased (0.1% per year) in trials using high doses, probably resulting from overdosing and related Omega‑3 Indices > 16% [4]. Both safety issues underscore the need to determine the Omega‑3 Index in a standardised manner, in this case to maximise safety.

Typical issues in tolerability are fishy hiccups and gastro-intestinal disturbances [13]. These symptoms can be minimised—and the bioavailability can be maximised—by ingesting the omega‑3 supplement with the main meal of the day [13].

## Other fatty acids

Of the remaining 24 red blood cell FAs determined with the standardised method in erythrocytes, many have been proven to be important. Adding 3 other FAs (14:0, 16:1n‑7 and 22:0) to the Omega‑3 Index was found to be as predictive of total mortality as a combination of standard risk factors [24]. Levels of industrially produced trans-FAs have been found to be low in Germany and safe at levels < 1.04% [44]. In the US, these trans-FA levels declined in parallel with fatal ischaemic heart disease rates through the years and are now at safe levels [45]. In contrast, dairy-derived trans-FA levels were found to be associated with reduced total mortality [44].

Of the saturated FAs measured, only 16:0 (largely derived from endogenous de-novo lipogenesis) was positively associated with increased total mortality, while the other saturated FAs (14:0, 18:0, 20:0, 22:0 and 24:0) were not [46]. The monounsaturated FAs 18:1n‑9, 20:1n‑9 and 24:1n‑9 were positively associated with total mortality [47]. Of the omega‑6 FAs, 22:4n‑6 and 22:5n‑6 were positively associated, 18:2n‑6, 18:3n‑6 and 20:3n‑6 were inversely associated, but 20:4n‑6 (arachidonic acid) was not associated with total mortality [48]. Results obtained in other FA compartments were largely uninformative (e.g. [9, 10, 49]).

Thus, FA analyses can provide important information beyond EPA and DHA, with results obtained with the standardised analysis of erythrocytes painting the most detailed picture. This picture is incompatible with a group-wise nomenclature of FAs (e.g. ‘saturated’, ‘mono-unsaturated’ or ‘omega-6’) and therefore opens a new chapter in FA research. Clearly, the relevance of FA analysis beyond EPA and DHA also provides a strong argument for a standardised analytical procedure.

## Discussion

Once future epidemiological studies and intervention trials address the issues of study design discussed here, they will provide clearer answers to whether EPA and/or DHA are safe and effective in given health issues. The same will be true for clinical practice, since a targeted use of EPA and DHA based on the Omega‑3 Index will maximise efficacy and minimise untoward effects.

Focussing on omega‑3 status rather than intake provides a clear picture of the relevance, effects, safety and tolerability of EPA + DHA. This can be explained from various angles, starting with the fact that red blood cell EPA + DHA correlate with EPA + DHA in all other cells in the body thus far investigated. This perspective demands that the measurements be both standardised and integrated into clinical routine. Several laboratories, including large chains in Germany, have successfully adopted the standardised analytical method, demonstrating the feasibility and viability of this approach. Clearly, this would also be possible in the Netherlands. To become part of clinical routine, however, the Omega‑3 Index would also need to be reimbursed in the Netherlands, as it is in Germany. We feel the scientific data have matured sufficiently for the Omega‑3 Index to qualify for reimbursement.

A serious limitation is that laboratories provide results from other FA compartments (e.g. serum) or from non-standardised analyses of red blood cells that usually differ, sometimes substantially, from the results of standardised analyses of red blood cells [4]. Moreover, some laboratories relate the results they derive from non-standardised methods to the target range defined for the standardised analyses [4]. The consequence will be either over-ingestion or under-ingestion of EPA and DHA. Logically, in both cases, the target range of the Omega‑3 Index will not be reached. Since the Omega‑3 Index is a risk factor for total mortality and other serious clinical events, and chances of adverse effects (e.g. atrial fibrillation) can be minimised in the properly determined target range, providing results from non-standardised or harmonised analyses must be considered an ethical issue.

## Conclusion

Not using the Omega‑3 Index as a proxy for the omega‑3 status in previous intervention trials may at least partly explain their inconsistent results. The use of such a status/risk marker is supported by observations that, compared with lower Omega‑3 Indices, those in the target range of 8–11% see associated beneficial effects on total mortality, major adverse cardiac events and other cardiovascular issues, such as stroke (i.e. endpoints in the trials mentioned). Positive effects on brain function and other health issues are also promoted by target-range Omega‑3 Indices, and adverse effects such as bleeding and atrial fibrillation are minimised. The relevance of the Omega‑3 Index in both research and clinical medicine calls for a standardised analytical procedure, which is already established in several laboratories worldwide, and for a discussion on possible reimbursement of the Omega‑3 Index within national medical systems.
